# Cellular Uptake and Toxicological Effects of Differently Sized Zinc Oxide Nanoparticles in Intestinal Cells †

**DOI:** 10.3390/toxics9050096

**Published:** 2021-04-27

**Authors:** Anna Mittag, Christian Hoera, Alexander Kämpfe, Martin Westermann, Jochen Kuckelkorn, Thomas Schneider, Michael Glei

**Affiliations:** 1 Department of Nutritional Toxicology, Institute of Nutritional Sciences, Friedrich Schiller University Jena, Dornburger Straße 24, 07743 Jena, Germany; thomas.schneider@uni-jena.de (T.S.); michael.glei@uni-jena.de (M.G.); 2 German Environment Agency, Swimming Pool Water, Chemical Analytics, Heinrich-Heine-Straße 12, 08645 Bad Elster, Germany; christian.hoera@uba.de (C.H.); alexander.kaempfe@uba.de (A.K.); 3 Electron Microscopy Centre, Friedrich Schiller University Jena, Ziegelmühlenweg 1, 07743 Jena, Germany; martin.westermann@uni-jena.de; 4 German Environment Agency, Toxicology of Drinking Water and Swimming Pool Water, Heinrich-Heine-Straße 12, 08645 Bad Elster, Germany; jochen.kuckelkorn@uba.de

**Keywords:** apoptosis, Caco-2, cell cycle, cytotoxicity, genotoxicity, LT97, nanoparticles, zinc oxide

## Abstract

Due to their beneficial properties, the use of zinc oxide nanoparticles (ZnO NP) is constantly increasing, especially in consumer-related areas, such as food packaging and food additives, which is leading to an increased oral uptake of ZnO NP. Consequently, the aim of our study was to investigate the cellular uptake of two differently sized ZnO NP (<50 nm and <100 nm; 12–1229 µmol/L) using two human intestinal cell lines (Caco-2 and LT97) and to examine the possible resulting toxic effects. ZnO NP (<50 nm and <100 nm) were internalized by both cell lines and led to intracellular changes. Both ZnO NP caused time- and dose-dependent cytotoxic effects, especially at concentrations of 614 µmol/L and 1229 µmol/L, which was associated with an increased rate of apoptotic and dead cells. ZnO NP < 100 nm altered the cell cycle of LT97 cells but not that of Caco-2 cells. ZnO NP < 50 nm led to the formation of micronuclei in LT97 cells. The Ames test revealed no mutagenicity for both ZnO NP. Our results indicate the potential toxicity of ZnO NP after oral exposure, which should be considered before application.

## 1. Introduction

Zinc is an essential trace element that is involved in a variety of reactions in the human body as part of numerous enzymes and proteins. The bioavailability of dietary zinc depends on the nutritional constituents and the chemical form of the zinc compound [1]. Zinc is generally more bioavailable from organic salts; however, exposure to zinc nanomaterials may increase the bioavailability of inorganic zinc salts, such as zinc oxide (ZnO) [2]. This improved bioavailability of nano-sized trace elements is already used in farm animal husbandry for animal feed supplementation. ZnO nanoparticles (NP) have been advocated to promote animal growth, strengthen the immune system, and increase the survival of farmed animals [3,4]. However, feeding the animals with ZnO NP can lead to several consequences, such as a decreased bone density or eggshell thickness in laying hens. Furthermore, NP might accumulate in carcasses and can be consumed by humans with animal food products, while the excretion of ZnO NP by animals leads to an increased environmental pollution [5].

Metal oxides are the most commonly used nanomaterials [6], and ZnO NP are the most produced metal oxide NP [7]. ZnO NP have unique optical, electrical, and chemical properties and are mostly used for electronic components, cosmetics, and pharmaceuticals, among other applications [8,9]. ZnO NP have some beneficial characteristics for food packaging, such as photocatalytic activity, a high stability, and antimicrobial effects. Furthermore, they can improve mechanical, thermal, and gas barrier capacities when used as an additive in packaging [10,11]. Notably, ZnO NP are transferred from packaging materials to food, a phenomenon that results in direct oral human exposure to these NP [12].

In literature, there are a number of studies that have investigated the potential toxicity of ZnO NP, and a review by Zhu, Wu [8] highlighted that ZnO NP are potentially toxic to different cell lines (e.g., A549 lung epithelial cells or HepG2 liver cells) and animals, such as mice, rats, and common carps, through various mechanisms. This toxic impact is mainly mediated by the production of reactive oxygen species (ROS) and, subsequently, oxidative stress, lipid peroxidation, and cell membrane damage [8]. In vivo experiments with birds have shown that ZnO NP induce erythrocyte alterations, which indicates mutagenic and cytotoxic effects, even at low and thus environmentally relevant doses and short exposure times [13]. It is important to note that these toxic effects can arise from ZnO NP itself but can also result from dissolved zinc ions. It is already known that free zinc is toxic to humans at higher doses [14]. The underlying mechanisms of free zinc ions and ZnO NP are different and must be considered separately. Therefore, a zinc salt should be included as an ion control when investigating ZnO NP.

Due to their extensive beneficial properties, ZnO NP are widely used, including in many everyday products. Consequently, a direct or at least an indirect human exposure is likely inevitable. So far, there is no standardised analytical method for measuring NP concentrations in the environment to determine realistic human exposure levels. However, there are models that predict the concentration in surface water, air, plants, etc. using various parameters, such as the amount of NP released into the environment. It was estimated that, in 2014, sediments accumulated the most ZnO NP (7000 tons), followed by natural and urban soils (1400 tons) and landfills (1000 tons). It is estimated that ZnO NP concentrations in the environment will have tripled in 2020 [15]. Thus, the exposure to ZnO NP is steadily rising and represents a real problem [16]. An extensive toxicological assessment will therefore be needed. The available data on ZnO NP toxicology and its risks to humans are incomplete and heterogeneous. In addition, there are few data on oral intake, even though it is one of the main routes of exposure in the human body [17]. Therefore, the aim of our study was to investigate the cellular uptake and potentially toxic effects of ZnO NP using two human intestinal cell lines reflecting oral exposure.

## 2. Materials and Methods

### 2.1. Preparation and Characterisation of ZnO NP Dispersions

Two different ZnO NP (primary particle sizes of <50 nm and <100 nm, according to the manufacturer) were used, and ZnO NP stock dispersions (1 mg/mL or 12,290 µmol/L) were freshly made before each new experiment by mixing ZnO nanopowder (#544906 [18,19] and #677450 [20,21]) in Millipore filtered water and vortexing it for 30 s. The resulting dispersions were ultrasonicated in an ultrasonic bath (Elma Schmidbauer GmbH, Singen, Germany) for 5 min (ZnO NP < 100 nm; critical delivered sonication energy: 53.7 J/mL) or 10 min (ZnO NP < 50 nm; critical delivered sonication energy: 107.5 J/mL), before application. After sonication, the dispersions were vortexed again for 30 s.

The mean hydrodynamic diameter and polydispersity index of ZnO NP were measured via dynamic light scattering (polystyrene cuvette, Sarstedt AG & Co. KG, Nümbrecht, Germany), and the zeta potential was measured using electrophoretic mobility (Zetasizer Nano ZS with folded capillary cell DTS1060; Malvern Panalytical GmbH, Kassel, Germany). Furthermore, the ZnO NP size and shape were investigated using transmission electron microscopy (TEM; Carl Zeiss AG, Oberkochen, Germany). Therefore, freshly prepared ZnO NP dispersions (12,290 µmol/L) were placed on Formvar-carbon mesh grids (Quantifoil Micro Tools GmbH, Großlöbichau, Germany), dried, and analysed using a Zeiss CEM 902 A electron microscope (Carl Zeiss AG, Oberkochen, Germany). The specific surface area of ZnO NP was measured with nitrogen adsorption by the Brunauer-Emmett-Teller (BET) method.

The solubility of ZnO NP was investigated using inductively coupled plasma optical emission spectrometry (ICP-OES). ZnO NP stock dispersions were diluted to 1229 μmol/L with water or a cell culture medium. Samples were taken after 0, 1, 6, 24, and 48 h. They were centrifuged two times for 60 min at 5250× *g* (Allegra X-15R; Beckman Coulter GmbH, Krefeld, Germany). All samples were acidified to 2% with extra pure nitric acid (Suprapur Merck KGaA, Darmstadt, Germany). They were diluted at a ratio of at least 1:10 to exclude the matrix effects of a cell culture medium. Additionally, Y was added as an internal standard element. The total free zinc ions of the supernatant were determined by ICP-OES (iCAP™ 7000 equipped with an CETAC ASX-520 autosampler, both from Thermo Fisher Scientific Inc., Waltham, MA, USA) Mtwo different wavelengths: 206.2 nm and 334.502 nm. Two quality-check samples (QCs) in the concentration range of the analytical results were included and measured regularly after about 20 samples. The minimum number and the acceptance criteria for evaluating the QCs are consistent with the Food and Drug Administration guidelines [22]. The limit of quantification (on average 0.2 ± 0.1 mg/L) was determined after DIN 32645 [23]. The measurement of uncertainty was determined as 6.2%, according to DIN ISO 11352:2013-03, with five certified reference materials. Data processing was carried out with an inhouse developed program (from Chemical Analytics, German Environment Agency, Bad Elster, Germany), for quality management, as well as Qtegra™ (Thermo Fisher Scientific Inc., Waltham, MA, USA).

### 2.2. Cell Culture and NP Exposure

Two human colon cell lines of different origins and of different transformation stages were used for the cell culture experiments. The human colorectal adenocarcinoma cell line, Caco-2, represents an in vitro model of absorptive enterocytes for studying the absorption of nutrients and drugs [24]. They are functionally and structurally similar to the normal intestinal epithelium of the small intestine [25]. The Caco-2 cell line was obtained from ATCC (LGC Standards GmbH, Wesel, Germany). Caco-2 cells were cultured in Dulbecco’s Modified Eagle Medium, supplemented with 20% foetal bovine serum (FBS), 1% non-essential amino acids, and 1% penicillin/streptomycin (media and additives from Biochrom GmbH, Berlin, Germany). Cell passages between 20 and 60 were used. Additionally, the human colon adenoma cell line, LT97, which reflects an early stage of colon tumour development, was used. These cells were provided by Professor B. Marian from the Institute for Cancer Research at the University of Vienna in Austria. The LT97 cell line is based on cells from colon microadenoma of a patient suffering from hereditary familiar polyposis and can be regarded as a model reflecting early carcinogenesis [26]. The origin, properties, and culture conditions of LT97 cells have been described by Klenow, Pool-Zobel [27]. Passages between 10 and 25 were used. Both cell lines were cultured in an incubator (37 °C, 95% humidity, 5% CO_2_; Thermo Fisher Scientific Inc., Waltham, MA, USA) and tested for mycoplasma contamination at regular intervals. Both cell lines were verified by short tandem repeat profiling.

For the micronucleus analysis, 4′,6-diamidino-2-phenylindole (DAPI) assay, 3-(4,5-dimethylthiazol-2-yl)-2,5-diphenyl tetrazolium bromide (MTT) assay, and real-time cell analysis, both cell lines were seeded in 96-well plates. For this, 7.5 × 10^4^ Caco-2 cells were used per well. Since LT97 cells need cell–cell contacts to survive, the determination of the cell number was not possible before seeding. Therefore, LT97 cells were harvested from 75 cm^2^ cell culture flasks at about an 80% confluency and diluted with 5 mL of a cell culture medium. The resulting cell suspension was 20-fold diluted with the cell culture medium, and 100 µL was used per well for seeding. For the cell cycle analysis and apoptosis detection, cells were seeded in 6-well plates with 3 × 10^5^ Caco-2 cells and 100 µL of the abovementioned 5 mL LT97 cell suspension in 2 mL of a cell culture medium per well. To investigate the cellular uptake of ZnO NP, the cells were seeded in 75 cm^2^ cell culture flasks. One million Caco-2 cells and 1 mL of the 5 mL LT97 cell suspension were used. Seeded cells were grown to a confluency of ~70%. Subsequently, a cell culture medium was removed, and the cells were treated with ZnO NP diluted in a cell culture medium, with 10% FBS at 12–1229 µmol/L (equivalent to 1–100 µg/mL) or zinc chloride (ZnCl_2_) as a salt control in equimolar concentrations. The cell culture medium without NP served as a negative control. A solvent control was included (10% water) to exclude any possible effects caused by the solvent. Furthermore, positive controls were used to verify the suitability of the test operating conditions. The chosen ZnO NP concentrations correspond to that of Sohal, DeLoid [28], in which the daily human NP intake was converted to an equivalent in vitro dose. Remarkably, there is no information about daily intake of ZnO NP. It is often assumed to be comparable to titanium dioxide E171 (5.4 mg/kg bodyweight/day) and therefore similarly calculated. The estimated oral ZnO NP exposure dose was approximately 20 µg/mL or 246 µmol/L. To investigate any possible toxic effects of low doses and to determine critical toxic concentrations, we selected concentrations around that dose for our studies.

### 2.3. Cellular Uptake

The NP uptake by Caco-2 and LT97 cells was investigated using two different approaches. For the TEM analyses, the cells were incubated with a sub-toxic concentration of 123 µmol/L ZnO NP or ZnCl_2_ for 24 h. After the treatment, the cell culture medium was removed, and the cells were washed twice with phosphate-buffered saline (PBS) to remove NP residues from the cells. Glutaraldehyde (2.5%) was added for 1 h. The cells were washed twice with 100 mM of a cacodylic acid buffer (pH 7.2) for 15 min. Finally, the cells were scrapped from the flasks and further processed, as already described by Schneider, Westermann [29].

Additionally, energy-dispersive X-ray (EDX) spectroscopy analyses of ultrathin sections were performed with a Tecnai G2 transmission electron microscope (FEI Company, Hillsboro, OR, USA) at an acceleration voltage of 200 kV. The electron beam was operated in STEM mode. For the detection of zinc, a multipoint-EDX analysis of the sample sections was performed using an energy-dispersive X-ray spectrometer system, Quantax 200, with an XFlash 5030 detector (detector and software from Bruker Corporation, Leipzig, Germany). To achieve a higher detectability and visibility of ZnO NP, the concentration of osmium tetroxide in the post-fixation and contrasting step was decreased from 1% to 0.1% osmium tetroxide in a 0.1 M cacodylate buffer; therefore, the contrast of the cell structures is low, and the cellular membranes are nearly invisible in these sections.

To quantify the cellular ZnO NP uptake, inductively coupled plasma mass spectrometry (ICP-MS) was used. Caco-2 and LT97 cells were incubated with 123 µmol/L ZnO NP or ZnCl_2_ for 24 h. Cells were washed twice with PBS before harvesting. After counting the cell number, they were digested overnight with 65% nitric acid at room temperature. Afterwards, a sonication bath-assisted digestion was performed for 1 h at 50 °C. All samples were diluted with purified water to reach a final nitric acid concentration of 2% and filtrated to remove any remaining solid cell components. Finally, the ionic zinc content was measured using ICP-MS (iCAP™ RQ from Thermo Fisher Scientific Inc., Waltham, MA, USA, equipped with the 4DX prepFAST autosampler from ESI Elemental Service & Instruments GmbH, Mainz, Germany) by determining the mass signal *m*/*z* = 66 (which corresponds to the zinc isotope with a mass of 66 amu). An internal standard (2 ppb rhodium in 2% nitric acid) was continuously added to the samples online and simultaneously determined. Three QCs with different concentrations within the measurement range were included and measured regularly after about 20 samples. The QC evaluation is consistent with the Food and Drug Administration guidelines [22], and the limit of quantification (on average 3.1 ± 0.5 µg/L) complies with the DIN 32645 [23]. The determination of the measurement uncertainty (8% from two certified reference materials), data processing, and quality management were carried out in the same way as for ICP-OES, which has been described above under Section 2.1.

### 2.4. Cell Viability Assays

The cellular metabolic activity was investigated using MTT colorimetric staining. After a 3–72 h incubation period with ZnO NP, ZnCl_2_, or 0.05% Triton X-100 as the positive control, the cells were washed with PBS, treated with 0.5 mg/mL MTT (stock solution: 50 mg MTT powder (Merck KGaA, Darmstadt, Germany) in 10 mL of PBS; working solution diluted with a cell culture medium), and incubated for 3 h at 37 °C. The cell culture medium was then removed, and 200 µL of dimethyl sulfoxide (Merck KGaA, Darmstadt, Germany) was added to each well to solubilise the resulting formazan. The metabolic activity was determined by an absorbance measurement of the solubilised formazan at 570 nm with a microplate reader (reference wavelength: 630 nm; Synergy 2; BioTek Instruments, Inc., Bad Friedrichshall, Germany).

DAPI staining was used to determine the cell number. After treatment with ZnO NP ZnCl_2_ or 0.05% Triton X-100 as the positive control for 3–72 h, the cells were washed with PBS and fixed with methanol (Carl Roth GmbH + Co. KG, Karlsruhe, Germany) for 5 min. After 15 min (to allow for methanol evaporation), the cells were treated with 20 µM of DAPI (diluted in PBS; Merck KGaA, Darmstadt, Germany) for 30 min at 37 °C. A microplate reader (Synergy 2) was used to measure the fluorescence intensity of the stained DNA at an emission wavelength of 460 nm (excitation at 360 nm).

Real-time cell analysis (RTCA) was used to investigate the effect of ZnO NP or ZnCl_2_ on the cell impedance, which is influenced by different cellular parameters, such as the proliferation rate, cell number, size, and shape. Therefore, the cells were seeded onto 96-well E-plates (OLS OMNI Life Science GmbH & Co. KG, Bremen, Germany) and grown for 24 h. They were incubated with ZnO NP or ZnCl_2_ for 60 h. The impedance of each well was measured hourly using RTCA (xCELLigence; OLS). The change in impedance is represented as the dimensionless parameter cell index.

### 2.5. Apoptosis Detection

To quantify the amount of living, apoptotic, and dead cells, cell staining with annexin V-FITC and propidium iodide was used. After incubation with ZnO NP or ZnCl_2_ for 24 h, the cells were harvested, and the cell number was adjusted to 5 × 10^5^. The cells were treated according to the manufacturer’s instructions [30]. The CytoFLEX flow cytometer (Beckman Coulter GmbH, Krefeld, Germany) was used to measure 1 × 10^4^ events per sample. Data processing was carried out using the CytExpert software (Beckman Coulter GmbH, Krefeld, Germany).

### 2.6. Cell Cycle Analysis

After 24 h of treatment with ZnO NP and ZnCl_2_, the cells were washed with PBS and subsequently harvested. Cell cycle analysis was performed according to Pozarowski and Darzynkiewicz (2004). After adjusting the cell number to 5 × 10^5^ cells, the cells were fixed with 70% ethanol, washed with PBS, and incubated with a PI staining solution (containing 0.1% Triton X-100, 10 µg/mL propidium iodide, 100 µg/mL DNase-free RNase, and filled up with PBS) for 10 min at 37 °C. The cellular DNA content was measured using the CytoFLEX flow cytometer. For each sample, 1 × 10^4^ events were measured. Data analysis was performed using the CytExpert software. From the resulting cellular DNA content frequency histograms, the percentage of cells in the G1, S, G2/M cell cycle phase could be determined. Cells in G2/M have twice as much DNA as G1 cells. Cells in the S phase have more DNA than cells in G1 but less than cells in G2/M phase [31].

### 2.7. Genotoxicity Assay

Micronuclei, which represent chromosomal fragments as a result of DNA breakage or entire chromosomes, were examined using the micronucleus analysis kit [32], according to the manufacturer’s instructions. Caco-2 cells were incubated for 24 h and LT97 cells for 48 h with ZnO NP or ZnCl_2_ to allow most of the cells to divide, since micronuclei are formed during cell division. Vinblastine (25 ng/mL) and mitomycin C (2.5 µg/mL) were used as positive controls. After incubation, the plates were placed on ice for 20 min. A nucleic acid dye working solution was added, and the plates were placed on ice for 30 min under a light source to start the photolysis of ethidium monoazide. After removing the solution, complete lysis solution 1 (containing SYTOX^®^ Green nucleic acid stain and RNase) was added and incubated for 1 h in the dark at 37 °C. Finally, complete lysis solution 2 (containing SYTOX^®^ Green nucleic acid stain and green fluorescent beads) was added for 30 min at room temperature in the dark. The cells were measured using a flow cytometer (BD FACSCanto II; BD Biosciences, San Jose, CA, USA).

### 2.8. Mutagenicity Assay

To determine the potential mutagenicity of ZnO NP, the Ames test was performed, according to OECD [33]. The test strains were *Salmonella typhimurium* TA98 and TA100. The potential metabolic activation of ZnO NP was considered by adding a metabolising enzyme system (S9 mixture) from rat liver. Millipore water served as the negative control. Based on the recommendations provided by OECD [33], the manufacturer’s specifications (Trinova Biochem GmbH, Gießen, Germany), and an internal validation, the positive controls were bis(2-chloroethyl)-ammonium chloride for TA100 without the S9 mixture, 2-aminoanthracene for TA100 with the S9 mixture, 4,6-dinitro-o-cresol for TA98 without the S9 mixture, and daunomycin for TA98 with the S9 mixture. All substances were obtained from Molecular Toxicology Inc., Boone, NC, USA. The bacterial strains were grown overnight at 37 °C. For each plate, 100 µL of the bacterial suspension, with approximately 1−2 × 10^9^ bacteria per mL, was mixed well with 2.5 mL of top agar, followed by 100 µL of the test substances. The mixture was then poured onto Vogel-Bonner plates. For samples with the S9 mixture, 500 µL of S9, 2.0 mL of top agar, and 100 µL of the test substances were added. The test strains were incubated for 48 h at 37 °C. Finally, the revertant colonies were counted using an automatic, software-supported colony counter (Sorcerer Colony Counter; Instem plc, Staffordshire, UK). According to the OECD guidelines, each sample was analysed in duplicate (i.e., two plates) [33]. The mutagenic ratio (MR) was calculated with the following equation: MR = mean number of reverse mutants per plate of the sample/mean number of reverse mutants per plate of the negative control.

### 2.9. Statistical Analysis

Each assay was repeated with at least three independent experiments. The figures were created using GraphPad Prism 5 (GraphPad Software, San Diego, CA, USA). The results shown in the figures represent the mean and standard deviation. Data processing was performed using IBM SPSS Statistics Version 26 (IBM Corporation, Armonk, NY, USA). One-way analysis of variance with the Ryan-Einot-Gabriel-Welsh *post hoc* test was performed to identify significant differences between the untreated control and treated cells. The Ryan-Einot-Gabriel-Welsh post hoc test was chosen, because it is recommended for homogeneous variances and the same number of cases. In addition, the comparison methods based on Ryan’s method have a comparatively high power [34].

## 3. Results

### 3.1. NP Characterisation

The characteristics of the two utilised ZnO NP are summarised in Table 1. ZnO NP < 50 nm had a smaller average hydrodynamic diameter (d_DLS_; 198 nm in a cell culture medium and 205 nm in water), compared to ZnO NP < 100 nm (256 nm in a cell culture medium and 240 nm in water).

**Figure 1:**
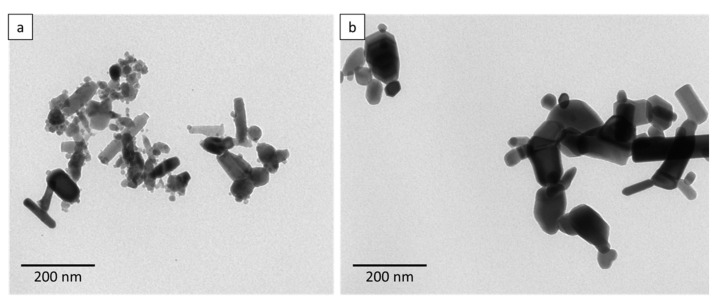
Representative transmission electron microscopy (TEM) images of ZnO NP < 50 nm (**a**) and ZnO NP < 100 nm (**b**) in a 1229 µmol/L dispersion in water.

This finding was confirmed by TEM images (Figure 1). Both dispersions contained larger, more rod-shaped particles, but also smaller, spherical particles.

Additionally, the dynamic light scattering intensity and the corresponding number distribution can be seen in Figure S1. The hydrodynamic diameter was around 200 nm for ZnO NP < 50 nm and 250 nm for ZnO NP < 100 nm. Since there were no large aggregates and, additionally, a low polydispersity, there were only marginal differences between the intensity and number distributions detectable. The long-term stability over a period of 72 h is represented in Figure S2. It can be seen that the NP size remained stable, especially in cell culture medium.

**Figure 2:**
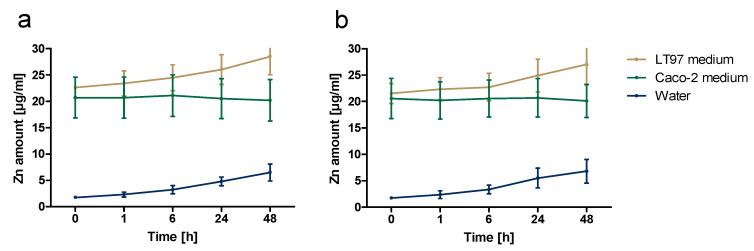
Dissolution behaviour of ZnO NP < 50 nm (**a**) and ZnO NP < 100 nm (**b**) in various media (1229 µmol/L), measured after 0–48 h using inductively coupled plasma optical emission spectrometry.

To investigate the dissolution behaviour of ZnO NP, the freshly prepared stock dispersions were diluted to 1229 µmol/L with water, Caco-2, and the LT97 cell culture medium. After 0, 1, 6, 24, and 48 h, samples were taken and centrifuged, and the zinc content was determined using ICP-OES (Figure 2). The dissolution behaviour of both ZnO NP was similar. Basically, the ZnO NP were more soluble in the cell culture medium than in water. The zinc ion amount in the solvent water increased for both ZnO NP from about 2% directly after the preparation to about 7% after 48 h. In the LT97 cell culture medium, the proportion of free zinc ions increased from about 22% up to 28.5% after 48 h. In contrast, the zinc ion content in the Caco-2 cell culture medium remained stable between 20–21% over time.

### 3.2. Cellular Uptake

**Figure 3:**
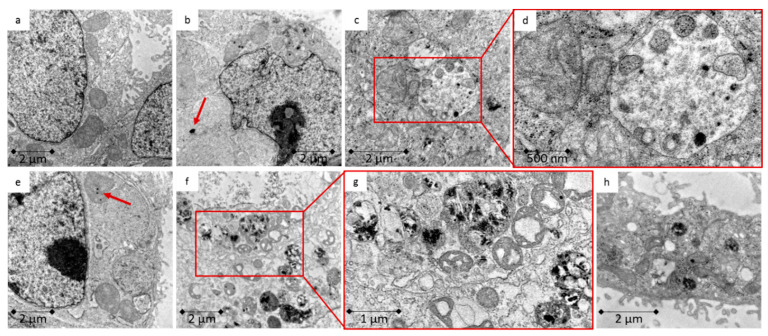
TEM images of Caco-2 cells; (**a**) untreated control; (**b**,**c**) 123 µmol/L ZnO NP < 50 nm for 24 h; (**d**) enlarged section of c; (**e**,**f**) 123 µmol/L ZnO NP < 100 nm for 24 h; (**g**) enlarged section from f; (**h**) 123 µmol/L ZnCl_2_ for 24 h; red arrows mark possible ZnO NP.

**Figure 4:**
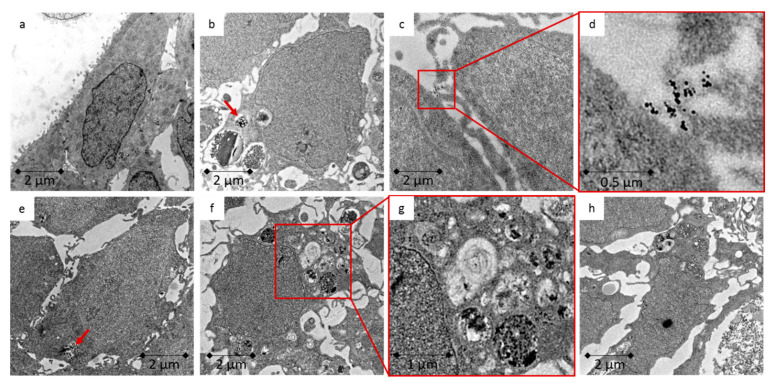
TEM images of LT97 cells; (**a**) untreated control; (**b**,**c**) 123 µmol/L ZnO NP < 50 nm for 24 h; (**d**) enlarged section of c; (**e**,**f**) 123 µmol/L ZnO NP < 100 nm for 24 h; (**g**) enlarged section from f; (**h**) 123 µmol/L ZnCl_2_ for 24 h; red arrows mark possible ZnO NP.

TEM was used to investigate whether the Caco-2 cells (Figure 3) or LT97 cells (Figure 4) take up ZnO NP, as well as the potential cellular areas in which they accumulate and the cellular consequences. Agglomerates, especially those encapsulated in endosomes, occurred in both cell types. Furthermore, treated cells showed morphological differences, compared to untreated cells. They contained more mitochondria, but these organelles were large and perforated. The images revealed that there was a myriad of densified heterochromatin in the nucleus, as well as signs of autophagy inside the cells.

EDX analyses were carried out on a selected, representative example to verify the ZnO NP. The results can be found in Figure S3. The particulate structures clearly show a zinc peak and are due to ZnO NP.

**Figure 5:**
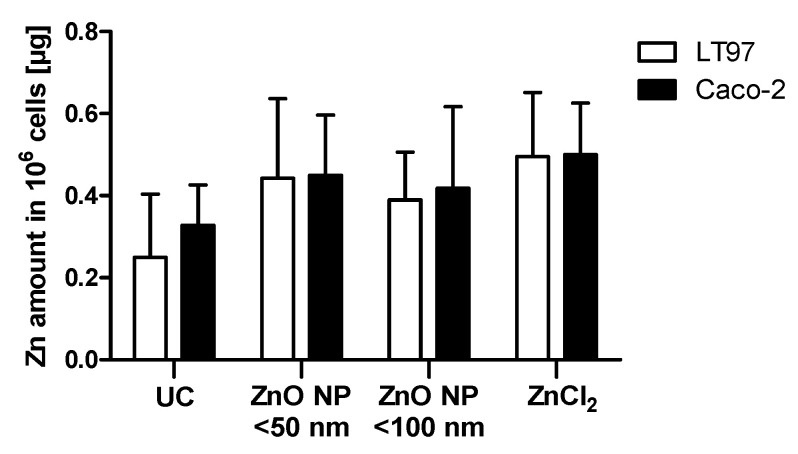
The cellular zinc content of LT97 and Caco-2 cells treated with 123 µmol/L ZnO NP < 50 nm, ZnO NP < 100 nm, or ZnCl_2_ for 24 h, measured with inductively coupled plasma mass spectrometry. UC = untreated control. The data are expressed as the mean + standard deviation; *n* = 4.

The cellular ZnO uptake was also investigated by measuring the total zinc amount using ICP-MS (Figure 5). The data are given for 10^6^ cells. The untreated LT97 cells contained 0.3 ± 0.2 µg zinc. Treatment with ZnO NP or ZnCl_2_ led to a slight but not significant increase of the cellular zinc amount (0.4 ± 0.2 µg zinc for ZnO NP < 50 nm; 0.4 ± 0.1 µg zinc for ZnO NP < 100 nm; 0.5 ± 0.2 µg zinc for ZnCl_2_). Similar results can be seen for the Caco-2 cells. The untreated control comprised 0.3 ± 0.1 µg zinc. Incubation with ZnO NP and ZnCl_2_ did not significantly increase the zinc amount (0.5 ± 0.1 µg zinc for ZnO NP < 50 nm; 0.4 ± 0.2 µg zinc for ZnO NP < 100 nm; 0.5 ± 0.1 µg zinc for ZnCl_2_).

### 3.3. Cytotoxic Effects on Caco-2 Cells

**Figure 6:**
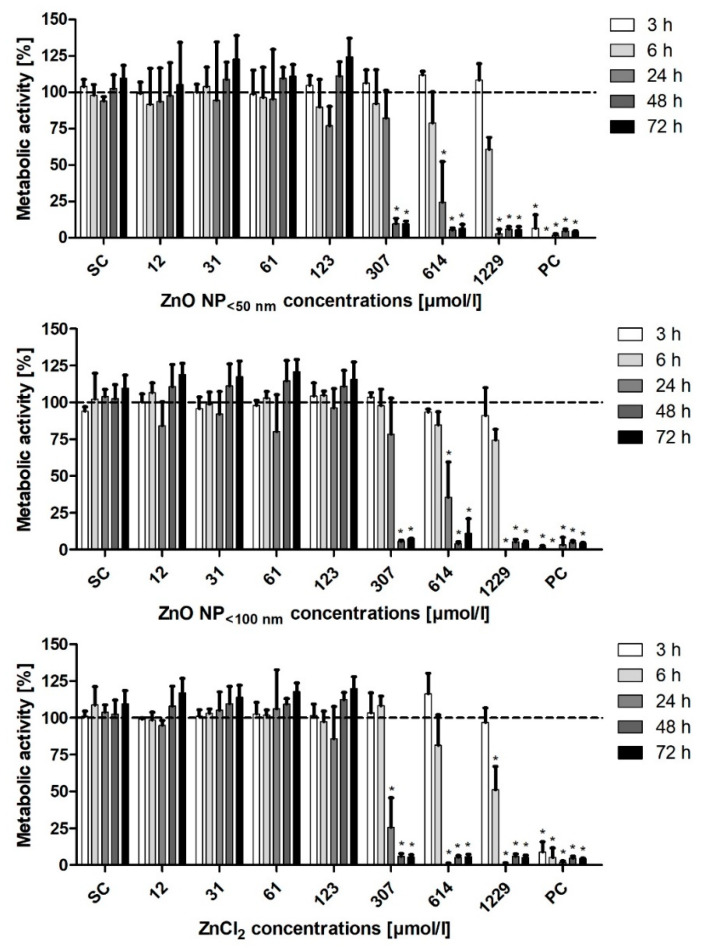
Relative metabolic activity of Caco-2 cells measured with the MTT assay, after treatment with ZnO NP or ZnCl_2_ for 3 h–72 h. SC: solvent control (Millipore water); PC: positive control (0.05% Triton X-100). The data are normalised to the untreated control (=100%; dashed line) and are expressed as the mean + standard deviation; *n* = 3. Significant differences compared to the untreated control (* *p*  ≤  0.05) were obtained by one-way analysis of variance/the Ryan-Einot-Gabriel-Welsh post hoc test.

Two assays were used to assess the cytotoxic potential of ZnO NP on Caco-2 cells. For both procedures, the data are normalised to the untreated control, which was set at 100%. The metabolic activity was investigated using the MTT assay (Figure 6). The metabolic activity of both NP and ZnCl_2_ decreased in a time- and concentration-dependent manner. The treatment of the Caco-2 cells with ZnO NP for 3 and 6 h did not significantly change their metabolic activity. After 24 h of treatment with 614 µmol/L ZnO NP, the metabolic activity declined significantly: ZnO NP < 50 nm: 24.6 ± 27.8%; ZnO NP < 100 nm: 35.6 ± 24% residual activity. ZnCl_2_ had a stronger effect on the Caco-2 cells, compared to ZnO NP. The metabolic activity was significantly reduced at 1229 µmol/L after 6 h of treatment (51.2 ± 15.8%). After 24 h, 307 µmol/L ZnCl_2_ significantly reduced the cellular metabolic activity (25.8 ± 20%).

**Figure 7:**
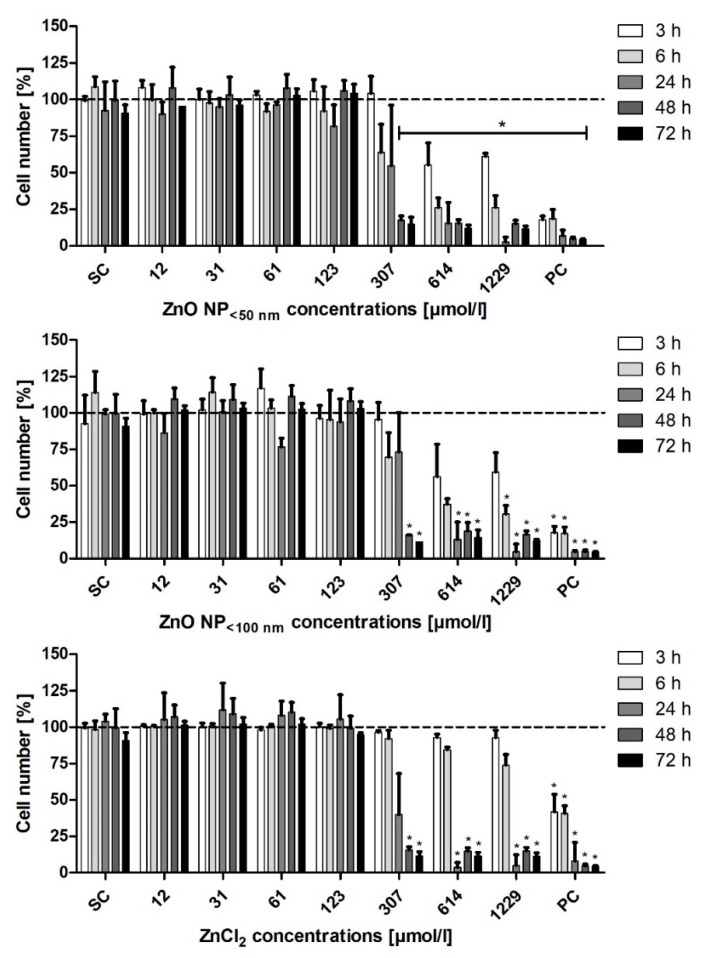
Relative cell number of Caco-2 cells measured with the DAPI assay, after treatment with ZnO NP or ZnCl_2_ for 3 h–72 h. SC: solvent control (Millipore water); PC: positive control (0.05% Triton X-100). The data are normalised to the untreated control (=100%; dashed line) and are expressed as the mean + standard deviation; *n* = 3. Significant differences compared to the untreated control (* *p*  ≤  0.05) were obtained by one-way analysis of variance/the Ryan-Einot-Gabriel-Welsh post hoc test.

The DAPI assay was used to examine the effects of ZnO NP on the cell number. There were concentration- and time-dependent effects (Figure 7). ZnO NP < 50 nm produced the strongest effects on the Caco-2 cell number. After only a 3 h treatment, there was a significant decrease in the number of cells at a concentration of 614 µmol/L (55.3 ± 15.2% residual cells). For ZnO NP < 100 nm, a reduction in the cell number was apparent after 6 h of treatment with 614 µmol/L (37.3 ± 3.8%), but it was only significant at 1229 µmol/L (30.5 ± 6%). After 24 h of incubation, the cell number decreased significantly at 614 µmol/L (13.2 ± 11.8%). ZnCl_2_ did not affect the Caco-2 cell number as much as ZnO NP. There was a significant decrease in the number of Caco-2 cells after 24 h of treatment with 614 µmol/L ZnCl_2_ (3.1 ± 4.1%).

The EC_50_ values were calculated via nonlinear regression/one phase exponential decay. The results can be seen in Table S1.

### 3.4. Cytotoxic Effects on LT97 Cells

**Figure 8:**
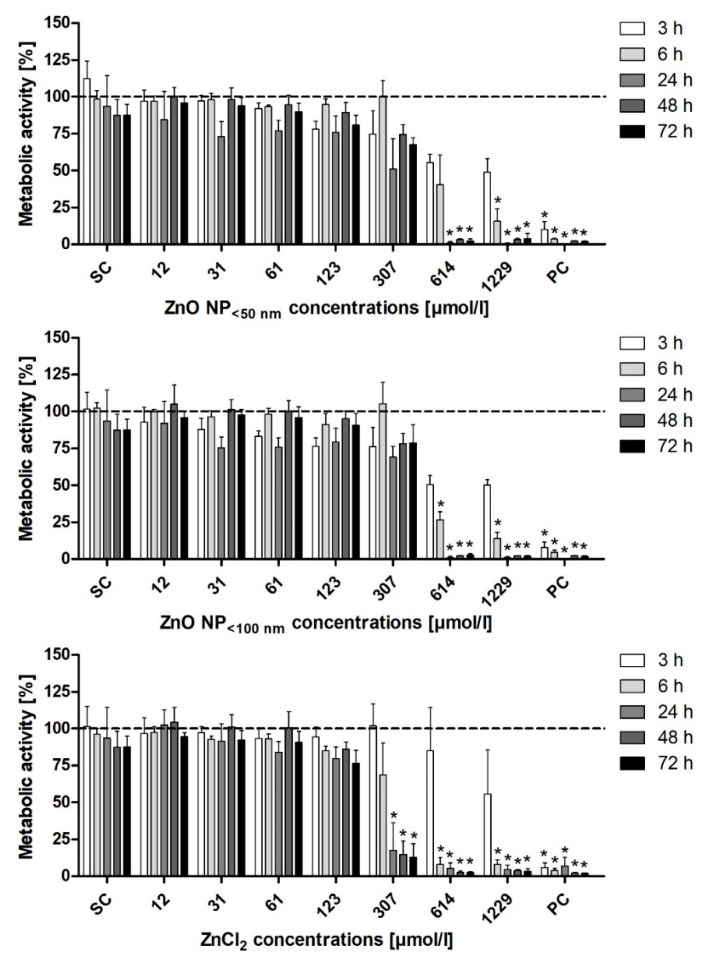
Relative metabolic activity of LT97 cells measured with the MTT assay, after treatment with ZnO NP or ZnCl_2_ for 3 h–72 h. SC: solvent control (Millipore water); PC: positive control (0.05% Triton X-100). The data are normalised to the untreated control (=100%; dashed line) and are expressed as the mean + standard deviation; *n* = 3. Significant differences compared to the untreated control (* *p*  ≤  0.05) were obtained by one-way analysis of variance/the Ryan-Einot-Gabriel-Welsh post hoc test.

Changes in the metabolic activity of LT97 cells were investigated using the MTT assay (Figure 8). Both ZnO NP (<50 nm and <100 nm) resulted in comparable effects. After 6 h of treatment, there was a significant reduction in the metabolic activity at 614 µmol/L ZnO NP < 100 nm (26.7 ± 5.3% residual activity) and at 1229 µmol/L ZnO NP < 50 nm (15.6 ± 8.5%). ZnCl_2_ already decreased the metabolic activity of the LT97 cells significantly after 6 h of treatment with 307 µmol/L (17.7 ± 18.6%).

**Figure 9:**
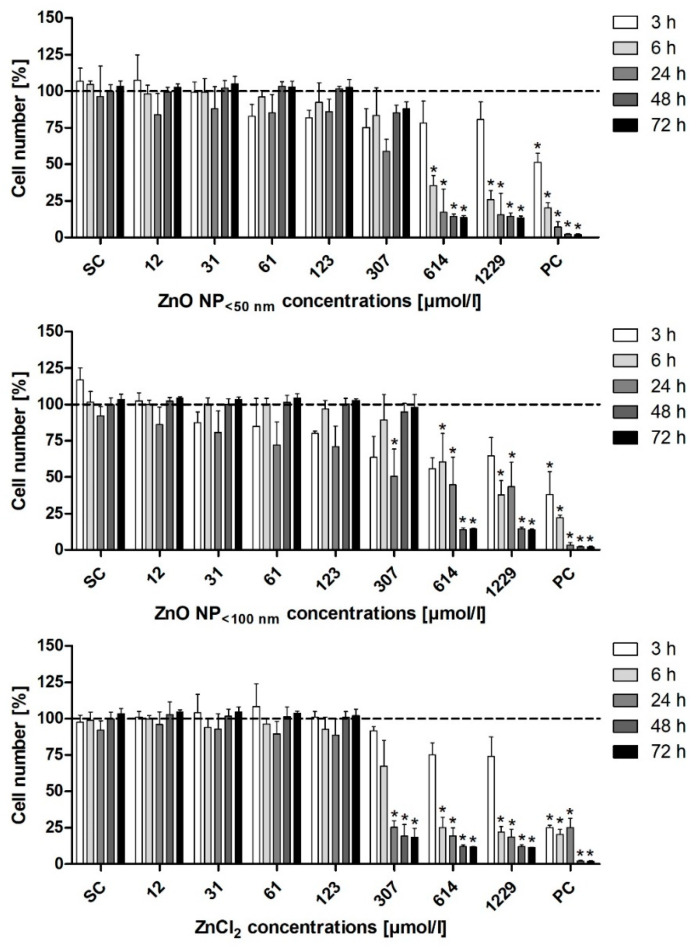
Relative cell number of LT97 cells measured with the DAPI assay, after treatment with ZnO NP or ZnCl_2_ for 3 h–72 h. SC: solvent control (Millipore water); PC: positive control (0.05% Triton X-100). The data are normalised to the untreated control (=100%; dashed line) and are expressed as the mean + standard deviation; *n* = 3. Significant differences compared to the untreated control (* *p*  ≤  0.05) were obtained by one-way analysis of variance/the Ryan-Einot-Gabriel-Welsh post hoc test.

The influence of ZnO NP on the LT97 cell number was examined using the DAPI assay (Figure 9). All three investigated zinc compounds showed concentration- and time-dependent effects. The treatment with ZnO NP < 50 nm significantly reduced the LT97 cell number after 6 h of incubation with 614 µmol/L (35.6 ± 6.8% residual cells). There was a significantly reduced cell number at 307 µmol/L after 24 h of treatment with ZnO NP < 100 nm (50.7 ± 18.7%) and ZnCl_2_ (25.4 ± 4.5%).

Altogether, ZnCl_2_ showed a stronger cytotoxicity to LT97 cells than ZnO NP, especially at a concentration of 307 µmol/L. The EC_50_ values were calculated via nonlinear regression/one phase exponential decay. The results can be seen in Table S1.

### 3.5. Real-Time Cell Analysis

**Figure 10:**
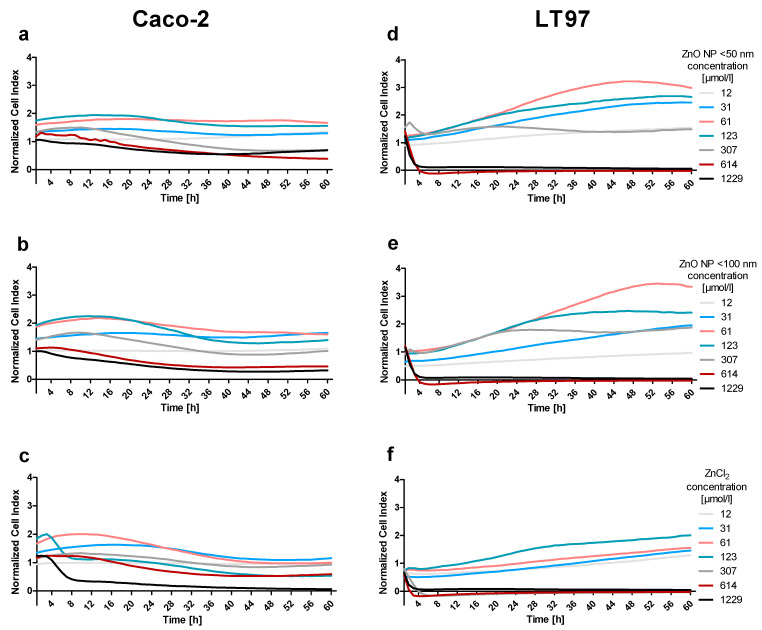
Real-time cell analysis of Caco-2 (**a**–**c**) and LT97 cells (**d**–**f**) exposed to ZnO NP < 50 nm (**a**,**d**), ZnO NP < 100 nm (**b**,**e**), or ZnCl_2_ (**c**,**f**), expressed as the cell index normalized to the untreated control, *n* = 3.

To investigate the real-time influence of ZnO NP and ZnCl_2_ on cellular parameters, such as the proliferation, size, and shape of Caco-2 and LT97 cells, the impedance-based assay RTCA was used (Figure 10). The data are expressed as the cell index normalized to the untreated control. In general, LT97 cells were more sensitive to all tested zinc compounds than Caco-2 cells. While concentrations of up to 123 µmol/L ZnO NP led to an increased cell index, there was a drastic cell index decrease in the LT97 cells after 3 h of treatment with 614 µmol/L ZnO NP (0.3 ± 0.2 for <50 nm; 0.2 ± 0.1 for <100 nm) and 307 µmol/L ZnCl_2_ (0.2 ± 0.03), compared to the untreated control. ZnO NP < 100 nm had a stronger influence on the cell index of the Caco-2 cells than ZnO NP < 50 nm. After 23 h of treatment with 1229 µmol/L, the cell index was reduced to 0.5 ± 0.4, compared to the untreated control. ZnCl_2_ turned out to be the most toxic zinc compound for Caco-2 cells. The cell index was reduced to 0.4 ± 0.2 after 10 h of treatment with 1229 µmol/L. To obtain a better overview, the EC_50_ values were calculated for incubation times of 24 h and 48 h via nonlinear regression/one phase exponential decay (Table S2).

### 3.6. Apoptosis Induction

**Figure 11:**
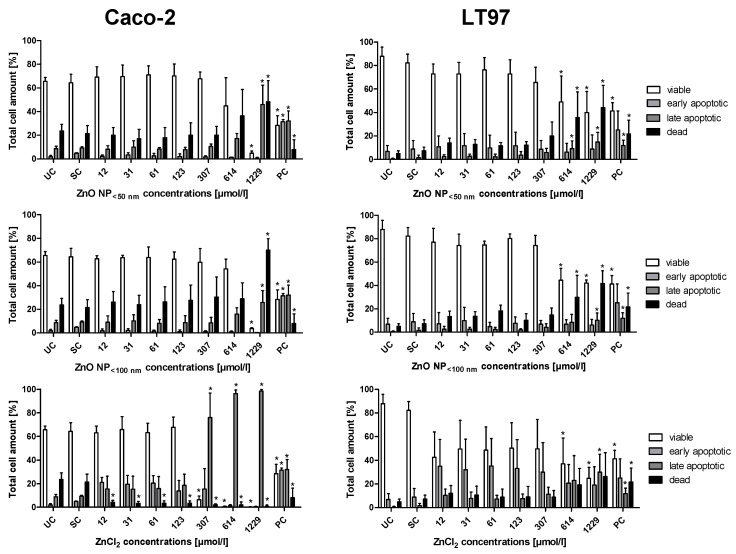
Proportion of viable, dead, early, and late apoptotic Caco-2 (**left**) and LT97 cells (**right**) after 24 h of treatment with ZnO NP < 50 nm, ZnO NP < 100 nm, or ZnCl_2_. UC: untreated control; SC: solvent control (Millipore water); PC: positive control (16 µM camptothecin). The data are expressed as the mean + standard deviation; *n* = 3. Significant differences compared to UC (* *p*  ≤  0.05) were obtained by one-way analysis of variance/the Ryan-Einot-Gabriel-Welsh post hoc test.

The influence of ZnO NP on apoptosis was investigated in Caco-2 and LT97 cells using Annexin V-FITC and PI staining (Figure 11). The untreated control population of Caco-2 cells consisted of 65.5 ± 3.4% viable cells (Annexin V-FITC and PI negative), 2 ± 1.1% early apoptotic cells (Annexin V-FITC positive, PI negative), 8.8 ± 2% late apoptotic cells (Annexin V-FITC and PI positive), and 23.7 ± 5.5% dead cells (Annexin V-FITC negative, PI positive). Both ZnO NP led to a significant reduction of viable cells (4.9 ± 1.8% for <50 nm; 3.8 ± 0.7% for <100 nm) and an associated increase in late apoptotic (46 ± 16.4% for <50 nm; 25.8 ± 10% for <100 nm) and dead cells (48.3 ± 18% for <50 nm; 70.1 ± 9.6% for <100 nm) at 1229 µmol/L. ZnCl_2_ had a stronger influence on the Caco-2 cells. The proportion of dead cells decreased significantly at all tested concentrations in favour of the apoptotic cells. At a concentration of 307 µmol/L, the proportion of viable cells was significantly reduced (6.5 ± 3.2%), while the proportion of late apoptotic cells significantly increased (76 ± 20.7%).

The untreated control of LT97 cells comprised 87.9 ± 7.8% viable cells, 6.8 ± 5.1% early apoptotic cells, 0.5 ± 0.5% late apoptotic cells, and 4.9 ± 2.4% dead cells. The treatment with 614 µmol/L ZnO NP or ZnCl_2_ led to significant shifts in the proportions (48.9 ± 22% viable, 6.2 ± 7.4% early apoptotic, 9.3 ± 6.4% late apoptotic, and 35.6 ± 21.8% dead cells for ZnO NP < 50 nm; 54.9 ± 22.2% viable, 6.8 ± 3.7% early apoptotic, 8.4 ± 6.8% late apoptotic, and 29.9 ± 18.7% dead cells for ZnO NP < 100 nm; 37 ± 21.8% viable, 20.7 ± 15.7% early apoptotic, 23 ± 20.8% late apoptotic, and 19.2 ± 13.9% dead cells for ZnCl_2_).

### 3.7. Cell Cycle

**Figure 12:**
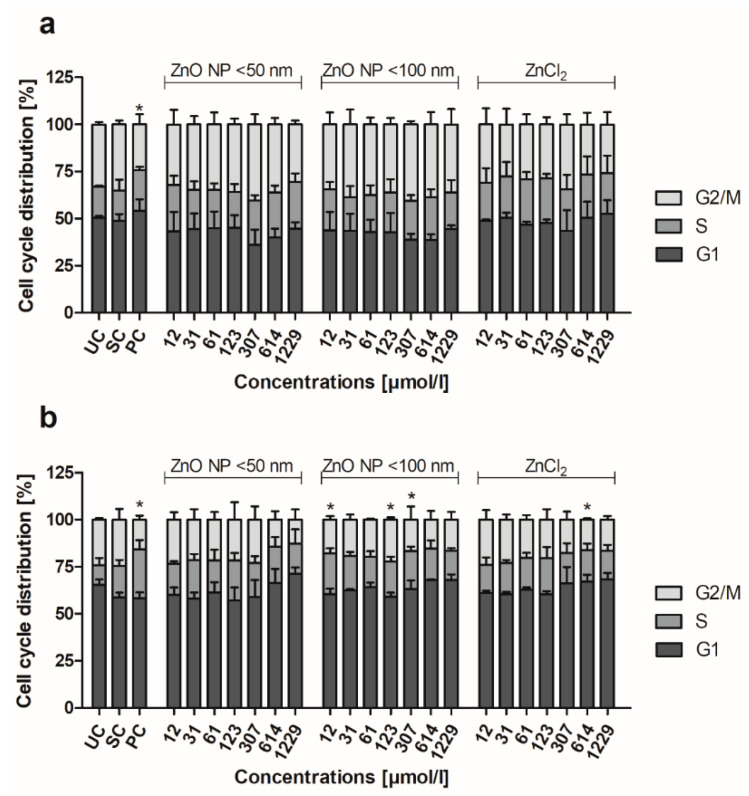
Proportion of Caco-2 (**a**) and LT97 cells (**b**) in the G1, S, and G2/M cell cycle phases, after 24 h of treatment with ZnO NP < 50 nm, ZnO NP < 100 nm, or ZnCl_2_. UC: untreated control; SC: solvent control (Millipore water); PC: positive control (16 µM camptothecin). The data are expressed as the mean + standard deviation; *n* = 3. Significant differences compared to UC (* *p*  ≤  0.05) were obtained by one-way analysis of variance/the Ryan-Einot-Gabriel-Welsh post hoc test.

Cell staining with propidium iodide was used to examine the cell cycle distribution of Caco-2 and LT97 cells. In the untreated control of the Caco-2 cells (Figure 12a), 50.5 ± 1% of the cells were in the G1, 16.4 ± 0.6% in the S, and 33.1 ± 1.2% in the G2/M phase of the cell cycle. The ZnO NP and ZnCl_2_ treatment did not significantly alter these proportions.

The untreated control of the LT97 cells comprised 65.4 ± 2.9% cells in the G1, 10.5 ± 3.7% cells in the S, and 24.1 ± 1% cells in the G2/M phase of the cell cycle (Figure 12b). The treatment with ZnO NP < 50 nm did not significantly change the proportions. The incubation with 12, 123, and 307 µmol/L ZnO NP < 100 nm resulted in significantly more cells in the S phase (21.8 ± 2.7%, 18.6 ± 2.7%, and 20.2 ± 2.4%, respectively). In contrast, 614 µmol/L ZnCl_2_ led to significantly fewer cells in the G2/M phase (16.1 ± 0.7%).

### 3.8. Genotoxic Effects

**Figure 13:**
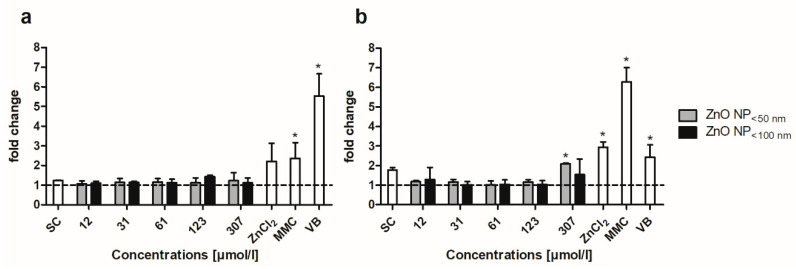
Micronuclei of Caco-2 (**a**) and LT97 cells (**b**) after treatment with ZnO NP < 50 nm and <100 nm for 24 and 48 h, respectively. SC: solvent control (Millipore water); MMC: 2.5 µg/mL mitomycin C; VB: 25 ng/mL vinblastine; 307 µmol/L ZnCl_2_ for Caco-2 cells and 12 µmol/L ZnCl_2_ for LT97 cells. The data are normalised to the untreated control (=1; dashed line) and are expressed as the mean + standard deviation; *n* = 3. Significant differences compared to the untreated control (* *p*  ≤  0.05) were obtained by one-way analysis of variance/the Ryan-Einot-Gabriel-Welsh post hoc test.

Micronucleus analysis was performed to examine whether ZnO NP caused chromosome damage or damage to the spindle apparatus. The data are normalised to the untreated control, which was set at 1. For both cell lines, only concentrations up to 307 µmol/L are shown; at 614 µmol/L and 1229 µmol/L, the cytotoxicity was too high, and there were no cells that could be evaluated. There was no significant increase detectable in the number of micronuclei in the Caco-2 cells (Figure 13a). In contrast, the LT97 cells were more sensitive to the ZnO NP treatment. There was a significant increase in the number of micronuclei in the LT97 cells at 307 µmol/L ZnO NP < 50 nm (Figure 13b). For both cell lines, the ZnCl_2_ treatment led to the most prominent effects. The most notable effect was triggered by 12 µmol/L ZnCl_2_, which significantly increased the number of micronuclei in the LT97 cells. The effect of zinc ions seems to ignite genotoxic effects much more effectively than ZnO NP. These findings could also be tendentially seen in the Caco-2 cells.

### 3.9. Mutagenic Effects

The Ames test was performed to examine ZnO NP as a possible mutagen (Table 2). The mutagenicity of ZnO NP (<50 nm and <100 nm) and ZnCl_2_ was negative at all tested concentrations for both *S. typhimurium* strands, with and without S9 activation. In contrast, the used positive controls substantially increased the number of revertant mutants of *S. typhimurium*. The MR was less than 2 in all samples (except positive controls), confirming that ZnO NP and ZnCl_2_ are not mutagenic at all tested concentrations (Table S3).

## 4. Discussion

Zinc represents an essential structural component and regulator of various biological macromolecules and physiological cell events, all of which are important for normal human development and health [14]. Given that metal-based NP like ZnO NP negatively affect the activity, abundance, and diversity of flora and fauna, they must be precisely monitored [35]. ZnO NP have many beneficial properties, such as catalytic, anti-bacterial, and anti-inflammatory activities. They promote wound healing and can be used for bio-imaging [36]. The U.S. Food and Drug Administration has listed zinc as generally recognised as safe, because it is an essential micronutrient. However, this designation does not necessarily mean that ZnO NP can be considered safe [37]. There are some commercial food or food-related products that contain ZnO NP. Thus, they are directly added to food as additives or used for food packaging for a variety of applications [38].

The toxicity of ZnO NP depends on its properties, especially its shape and size [35]. Therefore, the characterisation of these NP is essential for toxicological comparison studies. The investigated NP had a specific surface area of 23.2 m^2^·g^−1^ for ZnO NP < 50 nm and 13.3 m^2^·g^−1^ for ZnO NP < 100 nm. The mean hydrodynamic diameter was 198 nm for ZnO NP < 50 nm and 256 nm for ZnO NP < 100 nm in a cell culture medium, which contains proteins that bind to the NP surface and prevent agglomeration. These values represent the zeta average, which should be considered as an intensity-based overall average. The ZnO NP dispersions consist of small primary particles and aggregates, as shown in the TEM images. They were not shaped uniformly, and the zeta potential showed that they were slightly negatively charged. ZnO NP are usually slightly negatively charged [39,40]. Previous studies have shown that these negatively charged ZnO NP are absorbed in larger amounts than positively charged particles [41]. The agglomeration behaviour and the diverse structures of ZnO NP have already been demonstrated [39,42,43,44]. They can appear as agglomerated spherules or particles with a rod-like shape [45], as hexagonal shapes with some agglomerates [46], as crystalline and polygonal particles [47], as crystallised and rod-shaped [48], or with a spherical shape [49].

The cellular uptake was investigated using TEM and ICP-MS. The TEM images of Caco-2 and LT97 cells indicated a ZnO NP uptake, regardless of their particle size. This uptake led to clear intracellular changes. The treated cells were perforated, and they exhibited more mitochondria and areas of autophagy. Intracellular changes occurred in both cell lines after treatment with ZnO NP and ZnCl_2_. Using EDX analysis, the presence of ZnO NP within the cells could be demonstrated. ICP-MS measurements showed that a 24 h treatment with 123 µmol/L ZnO NP or ZnCl_2_ induces a 2-fold increase in the cellular zinc amount, compared to an untreated control. Other studies have also investigated the cellular uptake of ZnO NP. After treating WAG cells, which originate from *Wallago attu* gill tissue, with 614 µmol/L ZnO NP for 24 h, particles were visible inside the cells, which was confirmed with EDX [50]. In the human monocytic cell line, THP-1, there was a detectable internalisation of ZnO NP in endosomes after treatment with 246 µmol/L for 6 h [51]. Ickrath, Wagner [52] found ZnO NP inside human mesenchymal stem cells (hMSC), and ZnO NP induced enhanced autophagy in JB6 Cl 41-5a mouse skin epidermal normal cells [53].

Cellular uptake depends on various physicochemical properties, including the size, surface charge, coating, and chemical composition of the used NP, as well as the concentration and exposure duration and the cell type itself. Small particles (<100 nm) are internalised by endocytosis. Other uptake routes are receptor-mediated diffusion through membrane pores and passive uptake via adhesive interactions [36]. In general, the uptake efficiency is estimated to be 15–20 times higher for NP, compared to their bulk counterparts. Further, a higher toxicity seems to be associated with smaller particles [54]. Inside the cell, NP can bind to membranes, proteins, and DNA [35]. It has already been shown that ZnO NP are taken up through endocytosis and become encapsulated in vesicles. The pH decreases within these vesicles, so the dissolution rate of ZnO NP increases rapidly, a phenomenon that causes lysosome destabilisation and, consequently, an increased intracellular release of zinc ions. This can result in cytotoxic cell damage [55].

The ZnO NP cytotoxicity was examined using MTT and DAPI assays. In Caco-2 and LT97 cells, the metabolic activity and cell number were reduced, depending on the concentration and time, but independent of the particle size. Up to 123 µmol/L ZnCl_2_ and ZnO NP, no cytotoxic effects were recognizable. Notably, 307 µmol/L ZnCl_2_ decreased the cellular Caco-2 and LT97 metabolic activity and cell number more than equimolar ZnO NP. Using RTCA, the time- and dose-dependent toxicity of ZnO NP, as well as a more toxic impact of ZnCl_2_, could be confirmed. There are comparable studies that have investigated the cytotoxicity of ZnO NP. A significantly reduced cell viability has already been demonstrated in Caco-2 cells after 24 h of treatment with differently sized ZnO NP (30, 90, and 200 nm) at concentrations of 154–1229 µmol/L [56,57,58]. In LoVo colon carcinoma cells, there was a time- and dose-dependent decrease of cell number and cell viability using cell counting with trypan blue and a tetrazolium colourimetric WST-1 assay for cytotoxicity evaluation [45]. After the incubation of CAL 27 human tongue cancer cells with ZnO NP (average particle size: 50 nm) for 24 h, there was a concentration-dependent decrease in viability (half maximal inhibitory concentration [IC_50_]: 307 µmol/L) using the CCK-8 assay [42].

Other studies have used cell lines that do not correspond to an oral uptake to investigate ZnO NP-dependent cytotoxicity. Nevertheless, the results have predominantly confirmed the cytotoxic potential of ZnO NP [39,40,46,51,52,54,59,60,61,62,63,64,65,66].

In contrast, very few studies have reported a lack of cell damaging effects induced by ZnO NP. No cytotoxic effects were detectable in MG-63 and MDA-MB-231 breast cancer cells after treatment with 61–614 µmol/L ZnO NP for 24–72 h [43,67].

The release of zinc ions from ZnO NP, which we were able to show, could be followed by ROS induction and is often postulated as the main mechanism of ZnO NP cytotoxicity [45]. Alterations of the normal intracellular zinc content may modify cellular processes and, consequently, affect DNA replication, DNA damage repair, the electron transport chain, or cellular homeostasis. The consequences can include altered protein activities, oxidative stress, and, eventually, cell death [55].

The trace element, zinc, is important for various metabolic processes in the human body. Due to their small size, NP can mimic the activity of biomolecules and can therefore also interfere in the cell cycle or apoptosis regulation [55]. We were able to show that ZnO NP lead to Caco-2 and LT97 cell death via the apoptotic pathway. While there were no changes in the cell cycle regulation of Caco-2 cells after the ZnO NP treatment, it led to a significant shift in the cell cycle phases of LT97 cells. A comparison with the literature confirms the different cell cycle effects caused by ZnO NP. The treatment of MCF-7 breast cancer cells with ZnO NP did not change the proportion of the cell cycle phases. Instead, there was a shift in the sub-G1 phase, which includes apoptotic cells. Most of these cells were in early apoptosis [68]. The incubation of human keratinocyte cells (HaCaT) resulted in a cell cycle arrest in the G2/M phase, with a concentration-dependent decrease in the Cyclin B1 expression. There was also a significant increase of late apoptotic cells [40]. Moratin, Scherzad [64] found that apoptosis and necrosis are responsible for the cell death of FaDu carcinoma cells after treatment with ZnO NP.

In the next step, we investigated the genotoxic potential of ZnO NP. Considering the different doubling times, the Caco-2 cells were incubated with ZnO NP and ZnCl_2_ for 24 h and the LT97 cells for 48 h. Interestingly, while no effect on the number of micronuclei was found in the Caco-2 cells, there was a significant increase in the number of micronuclei in the LT97 cells after exposure to 307 µmol/L ZnO NP < 50 nm. At higher doses, there were no more analysable cells. In both cell lines, ZnCl_2_ increased the number of micronuclei more than equimolar ZnO NP. This suggests that chromosome or spindle apparatus damages are more likely due to free zinc ions. Zijno, De Angelis [69] showed a significant time-independent increase of binucleated Caco-2 cells bearing micronuclei after treatment with 258 and 369 µmol/L ZnO NP (mean hydrodynamic diameter: 942 nm; zeta potential: −15.6 mV). After the treatment of lung cells (A549 and V79) with 123–614 µmol/L ZnO NP for 3–40 h, there was also a significant increase in the frequency of detectable micronuclei [46,70]. In the study of Yin, Casey [54], there were more micronuclei in the WIL2-NS cells subjected to 123 µmol/L medium- and large-sized ZnO NP (primary particle size: 78 and 147 nm, respectively), compared with the smallest particles (26 nm) and negative control. There were also increased micronuclei frequencies after ZnO NP exposure in lymphocyte cultures from heparinised human blood samples [71], THP-1 cells [51], human embryonic kidney (HEK293) cells, NIH/3T3 mouse embryonic fibroblast cells [49], WAG cells [50], and HaCaT cells [40].

In contrast, the administration of ZnO NP in animals has not yet been shown to increase the number of micronuclei. Male Swiss mice were exposed daily to up to 150 mg/kg ZnO NP for five days, and half of the animals were left unexposed for another five days to determine the aftermath of the ZnO NP treatment. The bone marrow micronuclei assessment revealed no more micronucleated polychromatic erythrocytes in both ZnO NP-treated groups, compared to the controls [72]. In another study, ZnO NP were applied orally in three doses with volumes of 10 mL/kg to outbred ICR mice. After the preparation and staining of the bone marrow, there was no detectable increase in micronucleated polychromatic erythrocytes [73].

Apart from the examination of the micronucleated cells, we were also interested in the investigation of the mutagenic potential of the utilised ZnO NP. Our study revealed no mutagenic effects of ZnO NP in *S. typhimurium* strands TA98 and TA100, with or without S9 activation, up to a concentration of 10 µg/plate, irrespective of the particle size. It is quite interesting to compare our results with those of other studies using bacterial reverse mutation tests. On the one hand, there were no changes in the numbers of reverse mutants in *S. typhimurium* TA97a, TA100, and *Escherichia coli* WP2 trp uvrA, with and without S9 activation, after 72 h of treatment with up to 1000 µg ZnO NP (<100 nm)/plate [74]. There was also no change in the number of revertant colonies, compared to the negative control and solvent control, in *S. typhimurium* TA98, TA100, TA1535, TA1537, and *E. coli* WP2 uvrA, after incubation with up to 5000 µg ZnO NP (20 nm or 70 nm, each positively and negatively charged)/plate in the presence and absence of S9 [73]. On the other hand, there was a maximal mutagenic response of *S. typhimurium* TA98, TA1537, and *E. coli* WP2 uvrA at a concentration of only 0.008 µg ZnO NP/plate with the S9 mixture, a concentration that is noticeably lower than the previously used ones. Interestingly, in this study, the mutagenicity decreased with increasing concentrations, and the effects were strain-specific. *S. typhimurium* TA100 and TA1535 showed no mutations [75].

In general, many studies have confirmed that ZnO NP exert toxic effects on mammalian cells, even at low concentrations. The median lethal dose (LC_50_) values were between 123 µmol/L and 1229 µmol/L. Much less significant toxic effects were detectable in bacteria with an IC_50_ of 7641 µmol/L [76]. Size, concentration, and contact time seem to be the most important impact factors, and the main mechanisms of ZnO NP toxicity appear to be ROS production and, subsequently, oxidative stress, lipid peroxidation, and cell membrane damage [8]. It has already been shown that ZnO NP exhibit a selective cytotoxicity to cancer cells [55,77]. In our studies, there was a stronger toxicity to LT97 adenoma cells, compared to Caco-2 carcinoma cells. It must be noted that cell lines can generally only be regarded as a model, and that the results cannot simply be transferred to living organisms, especially humans.

## 5. Conclusions

ZnO NP (<50 nm and <100 nm) are internalised by Caco-2 and LT97 cells, which leads to different cell-specific toxic effects, especially at concentrations of 614 µmol/L and 1229 µmol/L. A decrease in metabolic activity and cell number, as well as an increased rate of apoptotic and dead cells, can be observed in both cell lines. ZnO NP < 100 nm intervene in the cell cycle of LT97 cells and ZnO NP < 50 nm, leading to the formation of micronuclei in these cells, but not in Caco-2 cells. The Ames test reveals no mutagenicity for both ZnO NP. Overall, our results indicate the potential toxicity of ZnO NP after oral exposure, which should be considered before application.

## Figures and Tables

**Table 1:** Physicochemical properties of the ZnO powders/dispersions.

ZnO NP	SSA [m^2^/g]	d_BET_ [nm]	d_DLS_ [nm]		PDI		Zeta Potential [mV]	
			Medium	Water	Medium	Water	Medium	Water
<50 nm	23.2	<50	198 ± 2	205 ± 1	0.15 ± 0.02	0.25 ± 0.01	−8.1 ± 2.3	0.04 ± 1.01
<100 nm	13.3	<100	256 ± 2	240 ± 2	0.16 ± 0.02	0.13 ± 0.02	−6.7 ± 3.2	−0.10 ± 0.84

SSA: specific surface area (determined by the Brunauer-Emmett-Teller [BET] method); d_BET_: primary particle diameter (provided by the manufacturer); d_DLS_: mean hydrodynamic diameter (from the DLS intensity distribution (zeta average)); PDI: polydispersity index; SSA and d_BET_ were measured as nanopowder; d_DLS_, PDI, and zeta potential were measured as 1229 µmol/L ZnO NP dispersion in a cell culture medium and water after ultrasonication.

**Table 2:** Number of reverse mutants of *Salmonella typhimurium* TA100 and TA98 after treatment with ZnO NP or ZnCl_2_, with and without S9 activation. The data are expressed as the mean ± standard deviation; *n* = 3. Significant differences compared to the untreated control (* *p*  ≤  0.05) were obtained by one-way analysis of variance/the Ryan-Einot-Gabriel-Welsh post hoc test.

Compound	Dose [µg/Plate]	Number of Reverse Mutants/Plate [Mean ± SD]			
		TA100		TA98	
		without S9	with S9	without S9	with S9
ZnO NP_<50 nm_	0	148 ± 12	137 ± 37	30 ± 8	22 ± 3
	0.1	139 ± 11	121 ± 2	32 ± 2	22 ± 4
	0.25	128 ± 21	125 ± 11	30 ± 5	22 ± 1
	0.5	139 ± 34	130 ± 14	29 ± 4	26 ± 2
	1	131 ± 22	126 ± 9	30 ± 6	22 ± 2
	2.5	143 ± 25	146 ± 27	31 ± 6	25 ± 3
	5	148 ± 8	148 ± 26	27 ± 1	26 ± 7
	10	148 ± 18	145 ± 21	33 ± 9	29 ± 5
ZnO NP_<100 nm_	0	155 ± 34	170 ± 16	27 ± 1	27 ± 9
	0.1	155 ± 27	177 ± 12	28 ± 5	27 ± 7
	0.25	144 ± 43	159 ± 18	26 ± 2	28 ± 12
	0.5	143 ± 24	158 ± 22	29 ± 5	36 ± 21
	1	141 ± 36	172 ± 11	29 ± 2	38 ± 28
	2.5	144 ± 31	169 ± 12	28 ± 5	36 ± 26
	5	143 ± 13	156 ± 10	27 ± 1	40 ± 15
	10	143 ± 13	166 ± 24	25 ± 1	46 ± 23
ZnCl_2_	17	123 ± 2	131 ± 18	18 ± 9	21 ± 2
PC		1136 ± 14 *	3729 ± 95 *	1820 ± 547 *	688 ± 164 *

PC: positive controls (bis(2-chloroethyl)-ammonium chloride for TA100 without S9; 2-aminoanthracene for TA100 with S9; 4,6-dinitro-o-cresol for TA98 without S9; daunomycin for TA98 with S9).

## Data Availability

The data presented in this study are available on request from the corresponding author. The data are not publicly available as this is not common in our area.

## Supplementary Materials

The following are available online at https://www.mdpi.com/article/10.3390/toxics9050096/s1, Figure S1: Particle size determination by dynamic light scattering. The scattering intensity (a,b) and the number distribution (c,d) of 1229 µmol/L ZnO NP < 50 nm (a,c) and 1229 µmol/L ZnO NP < 100 nm (b,d) are shown. The data are expressed as the mean ± standard deviation; *n* = 3. Figure S2: Long-term stability of zinc oxide nanoparticle (ZnO NP) dispersions in a cell culture medium (a) and water (b) after ultrasonication, measured over a period of 72 h. The data are expressed as the mean ± standard deviation; *n* = 3. Figure S3: TEM images of LT97 cells with ZnO NP < 50 nm on the cell surface (a) and inside the cells (b) and EDX analysis of the labelled areas with associated spectra (1–6). Blue spectra indicate zinc on particulate structures (peak at 8.63 keV = Zn Kα); green spectra are reference areas, without zinc detection; peaks at 8.0 keV = Cu Kα from copper grids. Table S1: EC50 values of ZnO NP < 50 nm, ZnO NP < 100 nm, and ZnCl_2_ for Figure 4, Figure 5, Figure 6 and Figure 7, separately calculated for each treatment time and cell line; n. d. = not detectable. Table S2: EC50 values of ZnO NP < 50 nm, ZnO NP < 100 nm, and ZnCl_2_, calculated from real-time cell analysis data for Caco-2 and LT97 cells. Table S3. Mutagenicity of ZnO NP and ZnCl_2_ in *Salmonella typhimurium* TA100 and TA98 strains, with and without S9 activation. The results are presented as the mutagenic ratio and are expressed as the mean ± standard deviation; *n* = 3. Significant differences compared to the untreated control (* *p*  ≤  0.05) were obtained by one-way analysis of variance/the Ryan-Einot-Gabriel-Welsh *post hoc* test.
