# Supplementary material for: Cellular Uptake and Toxicological Effects of Differently Sized Zinc Oxide Nanoparticles in Intestinal Cells †
Source: Toxics. 2021 Apr 27;9(5):96. doi: 10.3390/toxics9050096 (PMC8146923; doi:10.3390/toxics9050096)
Supplement: Supplementary file 1 [file toxics-09-00096-s001.zip › toxics-1161707-supplementary.pdf]

Supplementary Materials: Cellular Uptake and Toxicological Effects of Differently Sized Zinc Oxide Nanoparticles in Intestinal Cells

Anna Mittag, Christian Hoera, Alexander Kämpfe, Martin Westermann, Jochen Kuckelkorn, Thomas Schneider and Michael Glei

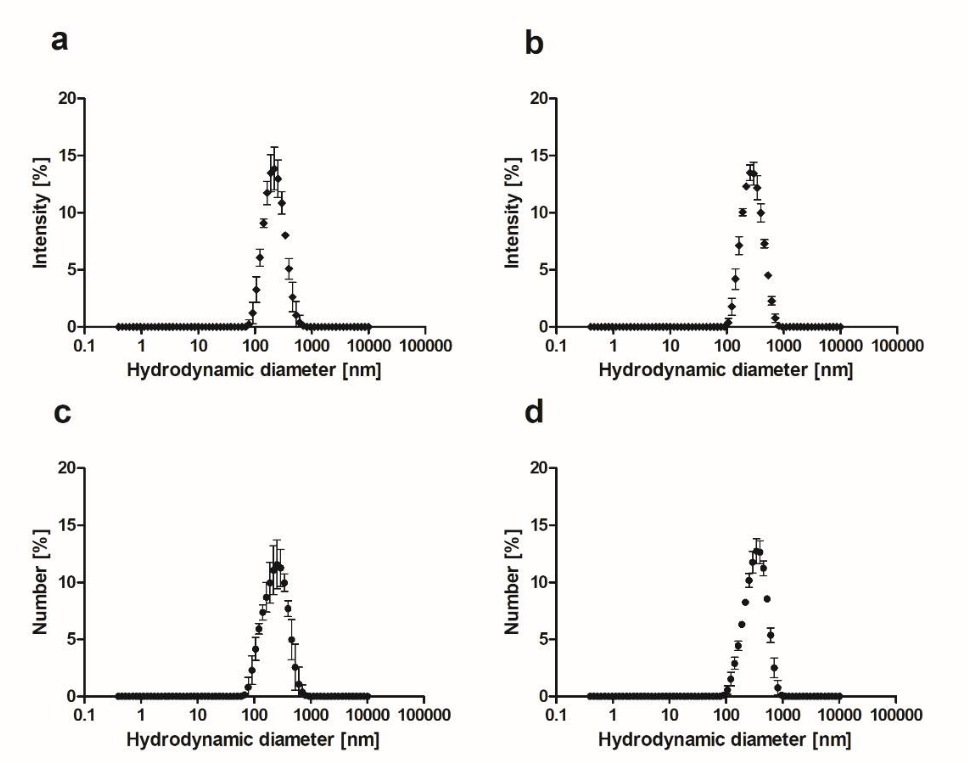

Figure S1. Particle size determination by dynamic light scattering. The scattering intensity (**a**, **b**) and the number distribution (**c**, **d**) of 1229 µmol/l ZnO NP <50 nm (**a**, **c**) and 1229 µmol/l ZnO NP <100 nm (**b**, **d**) are shown. Data are expressed as mean ± standard deviation; *n* = 3.

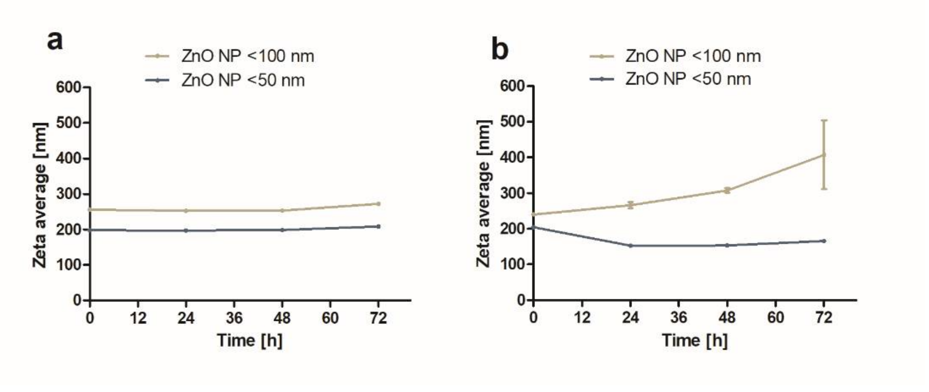

Figure S2. Long-term stability of zinc oxide nanoparticle (ZnO NP) dispersions in cell culture medium (a) and water (b) after ultrasonication measured over a period of 72 h. Data are expressed as mean ± standard deviation; *n* = 3.

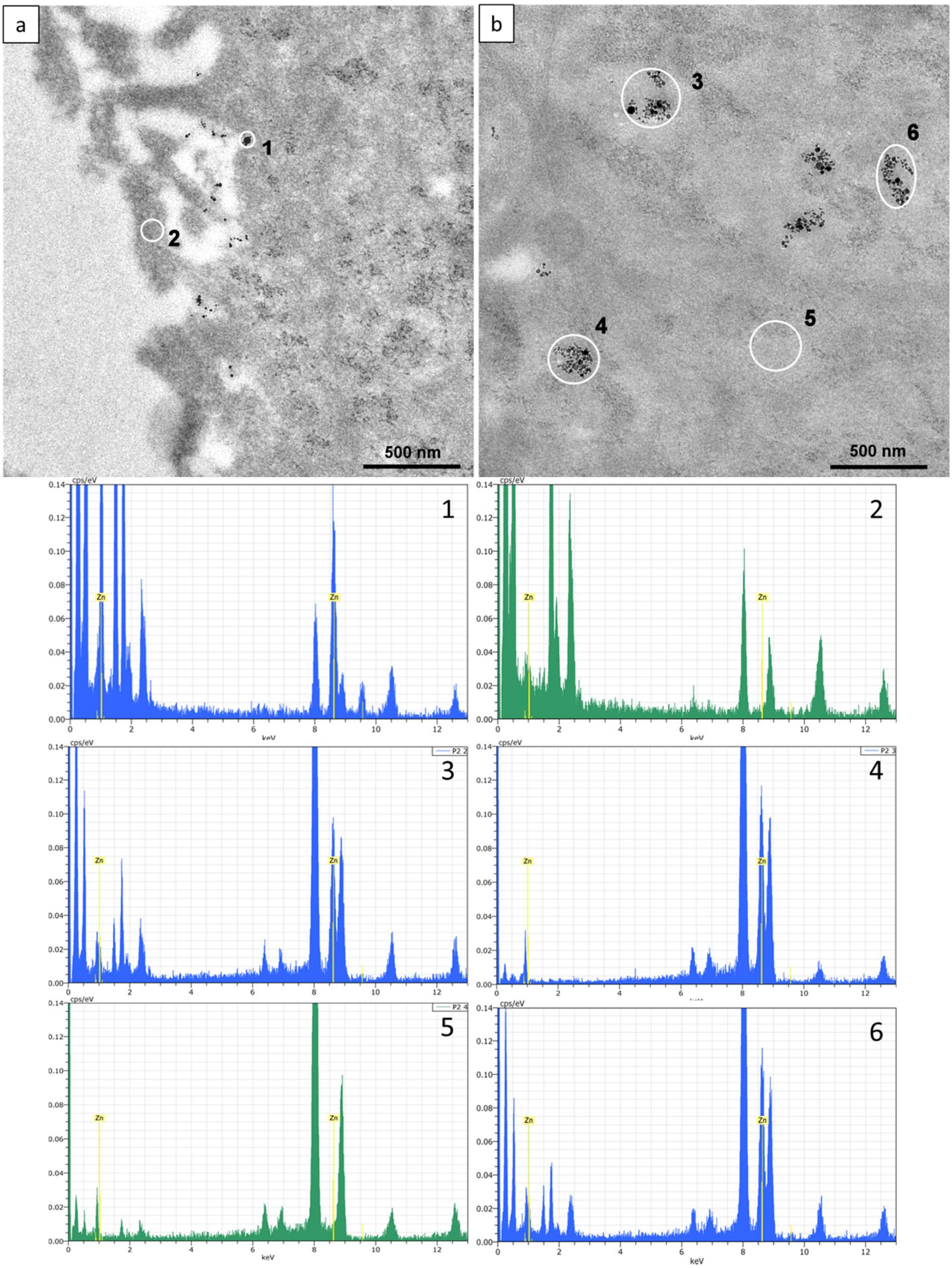

Figure S3. TEM images of LT97 cells with ZnO NP <50 nm on the cell surface (**a**) and inside the cells (**b**) and EDX analysis of labeled areas with associated spectra (1–6). Blue spectra indicate zinc on particulate structures (peak at 8.63 keV = Zn Kα); green spectra are reference areas without zinc detection; peaks at 8.0 keV = Cu Kα from copper grids.

Table S1. EC_{50} values of ZnO NP <50 nm, ZnO NP <100 nm and $ZnCl_2$ corresponding to figures 6–9, separately calculated for each treatment time and cell line; n. d. = not detectable (95% confidence interval in brackets).

Compound	Time [h]	EC_{50} [µmol/L]			
		Caco-2		LT97	
		MTT	DAPI	MTT	DAPI
**ZnO NP** **<50 nm**	3	n. d.	1286 (958–1874)	1028 (787–1393)	n. d.
	6	1602 (987–3947)	656 (455–909)	698 (552–873)	727 (562–932)
	24	509 (238–1005)	490 (298–706)	358 (190–541)	520 (320–750)
	48	240 (219–798)	537 (261–858)	520 (368–685)	628 (471–805)
	72	258 (251–849)	485 (190–824)	482 (327–647)	632 (476–808)
**ZnO NP** **<100 nm**	3	n. d.	1238 (876–1953)	1030 (767–1447)	1391 (835–3408)
	6	2122 (1659–2959)	755 (562–1006)	656 (493–852)	955 (783–1170)
	24	548 (363–762)	515 (324–733)	418 (251–599)	772 (368–1576)
	48	590 (349–883)	615 (387–888)	587 (452–737)	697 (549–869)
	72	582 (364–836)	571 (357–831)	566 (431–715)	708 (562–878)
**$ZnCl_2$**	3	n. d.	7341 (5151–13090)	1554 (1007–3210)	1887 (1378–2999)
	6	1333 (1032–1814)	2204 (1911–2607)	520 (354–706)	638 (471–839)
	24	467 (214–755)	527 (307–775)	401 (151–668)	506 (251–808)
	48	506 (233–820)	539 (278–843)	439 (200–697)	509 (265–784)
	72	532 (263–846)	483 (215–796)	379 (136–629)	513 (273–785)

Table S2. EC_{50} values of ZnO NP <50 nm, ZnO NP <100 nm and $ZnCl_2$ calculated from real-time cell analysis data for Caco-2 and LT97 cells (95% confidence interval in brackets).

Compound	Time [h]	EC_{50} [µmol/L]	
		Caco-2	LT97
**ZnO NP** **<50 nm**	24	1400 (712–6339)	803 (450–1485)
	48	1059 (485–4575)	823 377–1987)
**ZnO NP** **<100 nm**	24	1111 (615–2661)	735 (313–1660)
	48	852 (372–2239)	811 (292–2612)
**$ZnCl_2$**	24	1051 (349–6584)	418 (55–1019)
	48	644 (264–1224)	558 (121–1294)

Table S3. Mutagenicity of ZnO NP and $ZnCl_2$ in *Salmonella typhimurium* TA100 and TA98 strains with and without S9 activation. Results are presented as mutagenic ratio and are expressed as mean ± standard deviation; $n = 3$. Significant differences compared to untreated control (* $p \leq 0.05$) were obtained by one-way analysis of variance/Ryan-Einot-Gabriel-Welsh *post hoc* test.

**Compound**	**Dose [µg/plate]**	**Mutagenic Ratio**			
		**TA100**		**TA98**	
		**without S9**	**with S9**	**without S9**	**with S9**
$ZnO\ NP_{<50\ nm}$	0.1	1.0 ± 0.1	1.0 ± 0.1	1.1 ± 0.2	1.0 ± 0.1
	0.25	1.0 ± 0.1	1.0 ± 0.1	0.9 ± 0.1	1.1 ± 0.1
	0.5	1.0 ± 0.1	1.0 ± 0.1	1.1 ± 0.1	1.3 ± 0.2
	1	1.0 ± 0.1	1.0 ± 0.1	1.0 ± 0.1	1.2 ± 0.5
	2.5	0.9 ± 0.0	1.0 ± 0.0	1.0 ± 0.2	1.2 ± 0.4
	5	1.0 ± 0.3	0.9 ± 0.1	1.0 ± 0.1	1.3 ± 0.3
	10	1.0 ± 0.2	0.9 ± 0.1	0.9 ± 0.1	1.5 ± 0.4
$ZnO\ NP_{<100\ nm}$	0.1	1.0 ± 0.0	1.1 ± 0.1	1.1 ± 0.2	1.1 ± 0.1
	0.25	1.0 ± 0.1	1.0 ± 0.1	1.0 ± 0.1	1.1 ± 0.1
	0.5	1.0 ± 0.1	1.0 ± 0.1	1.1 ± 0.1	1.3 ± 0.3
	1	0.9 ± 0.0	1.0 ± 0.0	1.1 ± 0.1	1.3 ± 0.6
	2.5	0.9 ± 0.0	1.0 ± 0.0	1.1 ± 0.2	1.3 ± 0.5
	5	1.0 ± 0.4	0.9 ± 0.1	1.0 ± 0.0	1.5 ± 0.0
	10	1.0 ± 0.3	1.0 ± 0.1	1.0 ± 0.1	1.7 ± 0.3
$ZnCl_2$	17	0.9 ± 0.1	1.0 ± 0.1	0.7 ± 0.2	1.1 ± 0.2
PC		8.0 ± 1.1*	21.8 ± 2.1*	61.4 ± 2.4*	26.5 ± 3.0*

PC: positive controls (bis(2-chloroethyl)-ammonium chloride for TA100 without S9; 2-aminoanthracene for TA100 with S9; 4,6-dinitro-o-cresol for TA98 without S9; daunomycin for TA98 with S9).
